# A checklist to improve reporting of group-based behaviour-change interventions

**DOI:** 10.1186/s12889-015-2300-6

**Published:** 2015-09-25

**Authors:** Aleksandra J. Borek, Charles Abraham, Jane R. Smith, Colin J. Greaves, Mark Tarrant

**Affiliations:** Psychology Applied to Health group, University of Exeter Medical School, University of Exeter, St Luke’s Campus, Exeter, EX1 2LU UK

**Keywords:** Reporting guidelines, Reporting quality, Group-based interventions, Behaviour change interventions

## Abstract

**Background:**

Published descriptions of group-based behaviour-change interventions (GB-BCIs) often omit design and delivery features specific to the group setting. This impedes the ability to compare behaviour-change interventions, synthesise evidence on their effectiveness and replicate effective interventions. The aim of this study was to develop a checklist of elements that should be described to ensure adequate reporting of GB-BCIs.

**Methods:**

A range of characteristics needed to replicate GB-BCIs were extracted from the literature and precisely defined. An abbreviated checklist and a coder manual were developed, pilot tested and refined. The final checklist and coder manual were used to identify the presence or absence of specified reporting elements in 30 published descriptions of GB-BCIs by two independent coders. Reliability of coding was assessed.

**Results:**

The checklist comprises 26 essential reporting elements, covering intervention design, intervention content, participant characteristics, and facilitator characteristics. Inter-rater reliability for identification of reporting elements was high (95 % agreement, Mean *AC1* = 0.89).

**Conclusion:**

The checklist is a practical tool that can be used, alongside other reporting guidelines, to ensure comprehensive description and to assess reporting quality of GB-BCIs. It can also be helpful for designing group-based health interventions.

**Electronic supplementary material:**

The online version of this article (doi:10.1186/s12889-015-2300-6) contains supplementary material, which is available to authorized users.

## Background

Clear and comprehensive reporting of interventions is essential both to the advancement of behavioural science and the translation of research findings into improved service delivery [1]. Research syntheses using systematic review and meta-analytic methods can only accurately attribute differences in intervention effectiveness to particular intervention features if intervention descriptions are clear and comprehensive enough to highlight similarities and differences between interventions and allow features to be accurately categorized. Yet, the quality of scientific reporting of behavioural interventions remains inadequate [2, 3] creating problems for those reviewing research literature [4–9]. Similarly, replication and faithful implementation depends on accurate description of all intervention characteristics. However, studies assessing the quality and completeness of published descriptions of non-pharmacological treatments showed that over half of the assessed descriptions were not considered sufficient to allow replication of the interventions with procedures and materials being particularly poorly reported [10–12].

Acknowledgement of inadequate scientific reporting has resulted in the development of reporting guidelines [13], including the key standards, such as CONSORT (Consolidated Standards of Reporting Trials) for randomized controlled trials [14] and STROBE (STrengthening the Reporting of OBservational studies in Epidemiology) for observational studies [15]. Moreover, in recent years there has been an increasing focus on improving scientific reporting of the content of complex interventions [16, 17]. This has resulted in initiatives aimed at improving development and reporting of social, psychological and behaviour change interventions, such as the ITAX intervention taxonomy [18], CONSORT-SPI guideline (extension of CONSORT for social and psychological interventions) [19], TIDieR checklist (Template for Intervention Description and Replication) [20], and the work of the WIDER group (Workgroup for Intervention Development and Evaluation Research) [21]. Currently over two hundred published reporting guidelines are listed on the EQUATOR Network’s website [22], which was established to improve the reporting of health research.

Despite many initiatives to improve scientific reporting and evidence that reporting guidelines can improve the quality of reporting [23–28], complex interventions continue to be inadequately described [2]. Interventions vary widely and the meaning and application of terms used in guidelines may not apply equally well across intervention types. There is, therefore, a need for more specific standards that apply to particular types of interventions. Reporting guidelines, such as CONSORT, apply to a wide range of studies and include very general reporting suggestions. For example, CONSORT recommends that the description of interventions (item 5) should include ‘sufficient details to allow replication, including how and when they were actually administered’ ([14], p.699). However, this suggestion is unspecific with regard to what details are needed for replication with fidelity. TIDieR [20], further specifies some generic details needed for replication (e.g. materials, procedures, providers, modes of delivery, location, dose etc.). However, again, the meaning of these terms varies across intervention types and may be interpreted differently by researchers. Group-based interventions have particular features that need to be described to facilitate replication with fidelity. No existing reporting guidelines specify the details that are needed to replicate group-based interventions.

### Reporting of group-based behaviour-change interventions (GB-BCIs)

We define ‘group-based behaviour-change interventions’ (GB-BCIs) as behavioural interventions delivered by at least one facilitator to a group of (i.e. at least three) participants. Behavioural interventions can be based on educational and/or psychological change processes and techniques to facilitate changes in participants’ lifestyle or health-related behaviour, such as diet, exercise, smoking, drinking, condom use, self-management of chronic diseases etc. Many examples are available (e.g., [5, 29]). Scientific reporting of GB-BCIs may be especially challenging because many features of group context, composition and leadership influence how group participation impacts on individual behaviour [e.g., 30]. Research into group dynamics (e.g., [30, 31]) has identified a range of features that determine the effects of group participation on individual change. For example, group composition (i.e., who the group members are) can influence social identification [32], upward and downwards social comparisons [33], and group cohesion [34]. Activities engaged in by group members can facilitate particular types of learning including, for example, the use of social modelling [35]. Group leaders’, or facilitators’, background and facilitation style can shape interactions between members and, consequently, the personal impact of group participation [36]. These and many other characteristics, such as the time spent in groups and frequency of group meetings, that distinguish between different GB-BCIs need to be described to allow better understanding of change processes and the ‘active ingredients’ in groups and to allow more accurate replication.

Yet many published papers reporting evaluations of GB-BCIs do not provide even a basic characterization of the groups in the intervention. One systematic review comparing group and individual treatments for obesity found that details, such as, participants’ socio-economic status and facilitators’ training in intervention delivery and group facilitation, were frequently unreported [37]. Another systematic review of group-based diabetes self-management programs found that only five out of 11 included studies reported theoretical models used [29]. We confirmed poor reporting standards in a recent scoping systematic review identifying 126 papers reporting evaluations of group-based weight-loss interventions [38]. For example, group size was reported in 45 % of papers, a description of facilitators’ training was provided in 26 %, delivery-checks on fidelity were reported in only 10 % of articles. It was impossible to calculate the total group participation time for 37 % of these papers. Thus, although groups are commonly used in health-related behaviour-change interventions, papers reporting evaluations of such interventions often fail to explain why groups are used, how they are organized and delivered, how they work, and for whom they are most suitable [39]. Such ambiguity impedes accurate replication and the identification of reasons for between-study heterogeneity. This is important because meta-analyses of behavioural interventions commonly find substantial between-study heterogeneity in effectiveness. For example, a meta-analysis comparing 50 group-based weight-loss interventions with control groups showed that the mean difference in weight loss varied from -8.90 to 1.60 kg (with *I*^2^ = 89 %) (Borek A, Abraham C, Greaves C, Tarrant M. Group-based diet and physical activity interventions in overweight and obese adults: A systematic review and meta-analysis of randomised controlled trials. Forthcoming).

### The present research

We searched for guidelines on reporting of GB-BCIs and found only one useful framework [40]. Hoddinott et al. [40] articulate a series of questions that researchers should address when designing and evaluating GB-BCIs. These relate to the elements of intervention design, such as intervention setting, context, quantity, and mechanisms of change, to the elements of intervention delivery, such as facilitators’ delivery practices, and to the elements of participants’ and leaders’ attributes. Hoddinott et al. highlight the absence of guidelines for designing, evaluating and reporting group interventions in health research and call for further research. Their framework highlights the important elements of group interventions. In the work reported here we drew upon their findings and related work to develop and test a practical checklist that could be used by researchers to improve reporting and to evaluate the quality of reporting of GB-BCIs.

### Research questions

We addressed three questions:

1. What should be reported when describing GB-BCIs?
2. Can we develop a short, practical checklist identifying essential elements that should be included in reports of GB-BCIs?
3. Can such a checklist be used reliably to assess the content of intervention descriptions and quality of reporting in published reports of GB-BCI evaluations?

## Methods

In developing reporting recommendations for GB-BCIs we drew upon Moher et al.’s [3] guidance on developing reporting guidelines in health research. In particular, we used the following steps outlined by Moher et al. [3]: we identified the need for a guideline, reviewed the literature, generated a list of elements for consideration, discussed the rationale for inclusion of items in the checklist, pilot tested the checklist, and developed a guidance statement (the coder manual).

### What should be reported when describing GB-BCIs?

In order to identify reporting elements of GB-BCIs, firstly we selected 25 (20 %) reports of GB-BCI evaluations from the articles identified in the scoping review of group-based weight-loss interventions [38]. To ensure variation in the quality of descriptions, we randomly selected articles that had been stratified into two groups: those that were identified as comprehensive and partly comprehensive in the prior scoping review. Secondly, one of the authors (AB) read the reports, identified descriptions specific to delivery of interventions in a group setting, coded the descriptions in NVivo (version 9) and extracted quotes to illustrate the content of these descriptions. This emerging list of reporting elements was augmented with elements highlighted in Hoddinott et al.’s framework [40], elements relevant to group interventions included in other reporting guidelines [14, 15, 20, 41–43], and elements identified from the group dynamics literature [30, 31, 44]. The authors then discussed, named and defined each of the elements by providing questions to be addressed in the reports when describing each element. Finally the elements were grouped into themes related to the different aspects of GB-BCIs. This generated an extensive list of reporting elements, which would allow accurate replication of GB-BCIs. The extensive list is available as Additional file 1.

### Can we develop a short, practical checklist identifying essential elements that should be included in reports of GB-BCIs?

Since not all of the reporting elements included in the extensive list were applicable to all GB-BCIs, a further “essential elements” list was constructed by selecting only those elements that were expected to be relevant to all GB-BCIs. The authors discussed each element from the extensive list and reached a consensus on which should be retained as “universal” or “essential” in the sense that accurate reporting of such elements is required for replication of any GB-BCI. Each reporting element in the reduced “essential elements” list was named, defined, and categorized into the same themes as in the extensive list. Finally, the authors read and edited all reporting elements in the checklist until a consensus was reached about the inclusion and definitions of the checklist elements. The final checklist is available in Table 1.

**Table 1:** A checklist to improve reporting of group-based behaviour-change interventions

Reporting elements	Description	Reported: Yes / No
Intervention design		
1. Intervention source or development methods	Describes the source (origin) and/or methods used for developing the intervention.	
2. General setting	Reports the type of setting where the group sessions were delivered.	
3. Venue characteristics	Describes the set up or configuration of the room (or other venue) where the group meetings took place.	
4. Total number of group sessions	The total number of group sessions in the program is reported or it is possible for this to be calculated.	
5. Length of group sessions	Reports the length of group sessions (average and/or range).	
6. Frequency of group sessions	Reports the frequency of group sessions, i.e., how often they were delivered.	
7. Duration of the intervention	Reports the duration of the intervention, i.e., over what period of time the group sessions were delivered.	
Intervention content		
8. Change mechanisms or theories of change	Describes how the intervention was intended to work by identifying change mechanisms or underpinning theories of behaviour change.	
9. Change techniques	Describes the techniques used in group sessions to prompt change. These may be derived from the mechanisms or theories of change, and may use established taxonomies of behaviour change.	
10. Session content	Describes the content of the sessions in terms of themes or topics covered, i.e., what the sessions were about.	
11. Sequencing of sessions	Indicates whether there is a logical (sequential) progression of session content or, alternatively, that the content of all sessions is the same, i.e., a repetitive, or “rolling”, program with no particular start or end point.	
12. Participants’ materials	Reports what materials or tools the participants used during and outside the group sessions.	
13. Activities during the sessions	Describes what the participants and the facilitators did during group sessions, i.e., what happened during the sessions.	
14. Methods for checking fidelity of delivery	Reports methods used to check the fidelity of intervention delivery, i.e., methods used to check if the sessions were delivered as designed.	
Participants		
15. Group composition	Provides information on the composition of the groups in the intervention, i.e., who were the participants in the groups or whether there were any differences in the participants’ characteristics between groups.	
16. Methods for group allocation	Describes methods used to allocate the participants to different groups.	
17. Continuity of participants’ group membership	Indicates whether there was continuity in participants’ membership in a group throughout the program or if participants could switch between different groups.	
18. Group size	Reports the number of participants per group (average and/or range).	
Facilitators		
19. Number of facilitators	Reports the number of facilitators delivering the sessions, i.e., how many facilitators delivered each of the sessions.	
20. Continuity of facilitators’ group assignment	Indicates whether there was continuity in facilitator’s assignment to a group throughout the intervention, i.e., if the same or different facilitator(s) delivered the sessions to each group of participants.	
21. Facilitators’ professional background	Reports facilitators’ professional background, status as a non-professional, or relevant qualifications.	
22. Facilitators’ personal characteristics	Reports relevant personal characteristics of the facilitators, i.e., who they were in terms of age, gender, ethnic or cultural background, education level, socio-economic status etc.	
23. Facilitators’ training in intervention delivery	Reports what training in delivering the intervention the facilitators were provided with.	
24. Facilitators’ training in group facilitation	Reports what training in group facilitation methods the facilitators were provided with, i.e., how to work with and facilitate groups.	
25. Facilitators’ materials	Reports whether the facilitators were provided with materials and/or written instructions to be used to guide delivery of the sessions.	
26. Intended facilitation style	Describes the intended style of, or approach for, the session delivery and group facilitation.	

### Can the checklist be used reliably to assess the content and quality of reporting in published reports of GB-BCIs evaluations?

We tested how reliably the checklist could be used to identify characteristics in GB-BCIs in a two stage coding process.

First, the checklist was used by two authors (AB and JS) to independently code reporting elements in 25 (20 %) of the papers randomly selected from the scoping systematic review [38]. The coded elements were compared and inter-rater reliability was assessed as described below. This process highlighted ambiguities in some of the reporting elements’ definitions and differences in interpretation between the two coders. Differences and disagreements in coding between the two authors were used to refine the definitions of the reporting elements in the checklist and to create a coder manual to assist with identification of reporting elements. In addition to the named elements and definitions included in the checklist, the manual comprises (i) a brief introduction to the checklist, (ii) a series of general coding guidelines, (iii) practical coding tips, (iv) suggestions to aid identification of elements from descriptions, and (v) examples of text taken from GB-BCI descriptions illustrating instances of each reporting element. The final coder manual is available as Additional file 2.

Second, the checklist and the coder manual were used by the same two authors to identify reporting elements by coding 30 papers reporting evaluations of a range of GB-BCIs selected from six recent systematic reviews of group-based GB-BCI evaluations: five from a review of weight loss interventions [45], five from a review of physical activity interventions [46], 15 from reviews of self-management of chronic diseases (arthritis, asthma, and diabetes) [5, 6, 47], and five from a review of group-based smoking cessation interventions [48]. The reports were randomly selected, showed a wide range of quality of reporting, and included a range of target behaviours. Lists of the 25 papers selected for the first stage and the 30 papers selected for the second stage of testing are available from the first author.

We assessed the inter-coder reliability in identifying instances of defined characteristics in text using the “*AC1*” statistic proposed by Gwet [49]. Inter-coder reliability has been frequently assessed using Cohen’s kappa [50], which adjusts for the degree of agreement that can be expected to occur by chance alone. However, this statistic is sensitive to sample size and to category prevalence, especially, such as when there is a low prevalence of the defined category characteristics. This latter sensitivity can result in low kappa values even when agreement on presence or absence of a defined characteristic is very high. Gwet [49, 51] tested a series of reliability indices, including Cohen’s kappa, and concluded the *AC1* had the optimal set of output characteristics, providing a more realistic assessment of present/absent judgment agreement between two coders when the frequencies of some characteristics are low (see equations 7 and 8, p.5) [49].

## Results

### What should be reported when describing GB-BCIs?

A table with the extensive list of GB-BCI reporting elements we initially identified is available as Additional file 1. The 42 reporting elements, grouped into four themes: (i) intervention design, (ii) intervention content, (iii) participants, and (iv) facilitators, are presented with exemplary questions that should be addressed in the intervention descriptions in relation to each reporting element.

### Can we develop a short, practical checklist identifying essential elements that should be included in reports of GB-BCIs?

All 42 reporting elements identified in our extensive list *may be* relevant to any particular GB-BCI but we were able to identify 26 essential reporting elements which are relevant to *all* GB-BCIs. These 26 elements are categorized into the same themes describing: (i) intervention design, (ii) intervention content, (iii) participants, and (iv) facilitators. The checklist with the reporting elements and their definitions is presented in Table 1.

Below we describe the 26 elements briefly. We use the terms “participants” to refer to people receiving the intervention and “facilitators” to refer to people delivering or leading group sessions.

#### Intervention design

Seven reporting elements are specified to ensure replicability of the design of a GB-BCI. First, we recommend that researchers explain (1) how the intervention was developed and whether it was based on another group or individual intervention. If it is an original intervention we recommend that the intervention development methods are reported, for example, as specified by Intervention Mapping [52]. Second, (2) the setting in which the intervention took place including (3) venue characteristics, such as the layout and plan of the room in which group sessions took place, should be described. Finally, reports should detail time spent in groups by participants, including (4) the number, (5) length and (6) frequency of group sessions, as well as (7) the period of time over which the group meetings were held.

#### Intervention content

Seven reporting elements are specified to ensure replicability of GB-BCI content. We reiterate earlier calls for explicit use of (8) theory that specifies change mechanisms and, thereby, selection of change techniques [53]. (9) Mechanism-based change techniques used in interventions must be specified to ensure adequate replication [7, 45, 54–56]. Session plans should also be specified with descriptions including (10) content of each session, (11) any sequencing of session content, (12) descriptions of any materials used by participants, and (13) activities undertaken in the group sessions. Finally, (14) any methods used to check the quality and fidelity of session delivery should be reported. Fidelity of intervention and session delivery indicates the extent to which the intervention was delivered as intended and identifies any differences between intervention protocol and the actual delivery. Recommendations for ensuring and assessing fidelity have been published [57].

#### Participants

Four reporting elements are defined in relation to the characterization of participants. First, (15) the group composition and the characteristics of participants in the groups must be described and it should be made clear whether this was uniform or variable across intervention groups. Second, (16) the procedure by which groups were composed must be clarified. Were they, for example, formed purposefully, or opportunistically; in other words could participants select their group or, if not, how were they allocated? Similarly, it should be specified (17) whether participants were allocated to remain in the same groups (i.e., with the same participants) for the duration of the intervention or whether they could attend sessions across intervention delivered to different groups of participants. Finally, replication necessitates knowing (18) the group size.

#### Facilitators

Eight reporting elements are defined with regards to facilitator characteristics. Replication requires knowing (19) the number of facilitators delivering the sessions, (20) whether they changed or remained constant across group sessions and whether parts of a session were delivered by different facilitators - and if so why. For example, the majority of the sessions might be delivered by a generic facilitator but a session with a particular focus on diet might be delivered, or co-delivered, by a dietician. Reports should also include descriptions of facilitators’ characteristics including (21) their professional background, or their role as lay or peer leaders, as well as (22) their personal characteristics, such as their age, gender or cultural background. In addition, replication requires a detailed description of any training that facilitators received in (23) intervention delivery and in (24) group facilitation, and (25) what materials and instructions the facilitators used to deliver the sessions. Finally, a description of (26) the intended facilitation style will aid replication. This might include a simple indication of the extent to which the sessions were interactive, participant-driven or didactic, and the group processes and group atmosphere that the delivery style was meant to instigate.

### Can the checklist be used reliably to assess the content and quality of reporting in published reports of GB-BCIs evaluations?

In a pilot test of the initial version of the checklist AB and JS coded 25 papers reporting GB-BCIs evaluations. Coders achieved 86.6 % agreement (that is, agreement as to whether a reporting element was or was not included) across all 26 reporting elements. A mean *AC1* score of 0.73 was calculated. This level of agreement indicates an acceptable overall level of inter-rater reliability but four of the 26 reporting elements had inter-rater reliability scores below 0.7. Disagreements for these four elements were examined and discussed. Overall, trends of agreement and disagreement between the two coders were also considered. These discussions suggested that some checklist definitions could be refined and that a coder manual could be developed to further clarify the criteria by which coders should decide whether or not an intervention description adequately included each reporting element.

The refined and clarified checklist (as presented in Table 1) and the coder manual were then used by the same two coders to code a further 30 papers that included descriptions of GB-BCIs. Coders agreed in 94.5 % of cases generating a mean *AC1* score of 0.89. Agreement levels and *AC1* scores are presented for each reporting element in Table 2. All *AC1* scores for reporting elements exceeded 0.7, ranging from 0.72 to 1.00, indicating good inter-rater reliability for all 26 elements.

**Table 2:** Reliability in identification of essential reporting elements

Reporting elements	Number of agreements (% agreement)	AC1
1. Intervention source or development methods	28 (93)	0.891
2. General setting	29 (97)	0.934
3. Venue characteristics	30 (100)	1
4. Total number of group sessions	30 (100)	1
5. Length of group sessions	30 (100)	1
6. Frequency of group sessions	27 (90)	0.849
7. Duration of intervention	28 (93)	0.913
8. Change mechanisms or theories	25 (83)	0.748
9. Change techniques	30 (100)	1
10. Session content	30 (100)	1
11. Sequencing of sessions	27 (90)	0.802
12. Participants’ materials	27 (90)	0.849
13. Activities	29 (97)	0.961
14. Fidelity of session delivery	29 (97)	0.961
15. Group composition	30 (10)	1
16. Methods for group allocation	27 (90)	0.84
17. Continuity of participants’ group membership	24 (80)	0.723
18. Group size	28 (93)	0.872
19. Number of facilitators	29 (97)	0.934
20. Continuity of facilitators’ group assignment	29 (97)	0.937
21. Facilitators’ professional background	29 (97)	0.952
22. Facilitators’ personal characteristics	30 (100)	1
23. Facilitators’ training in intervention delivery	29 (97)	0.937
24. Facilitators training in group facilitation	29 (97)	0.958
25. Facilitators’ materials	26 (87)	0.76
26. Facilitation style	28 (93)	0.908
Total	737 (out of 780) (94.5 %)	0.892

## Discussion

### Summary

We have reported the development of a checklist of reporting elements needed to ensure comprehensive descriptions of GB-BCIs. An initial extensive list of 42 reporting elements, needed to adequately describe group-based intervention design, intervention content, group participants and group facilitators, was reduced to a checklist of 26 essential reporting elements that are expected to be common to all GB-BCIs. Building on a useful framework developed by Hoddinott et al. [40], the proposed checklist offers a practical and easy to use tool to encourage reporting of the elements critical to the understanding and replication of GB-BCIs.

Used in combination with a coder manual, our preliminary reliability testing found that all 26 defined reporting elements could be reliably identified, or marked as absent, in published papers. After a pilot refinement of the checklist definitions and the development of the coder manual, a second reliability test generated reliable judgments, with *AC1* scores of above 0.7 for all 26 reporting elements and agreement on more than 94 % of judgments.

### Strengths and limitations

The checklist and coder manual were tested on descriptions of GB-BCIs designed to promote weight loss, increase physical activity, enhance management of long term illnesses (including diabetes, arthritis and asthma) and support smoking cessation. We believe the checklist is relevant to the reporting of all GB-BCIs and, as such, provides a foundation for a common vocabulary for reporting that can be used to facilitate a better understanding of GB-BCIs and comparison of their design and delivery elements.

Our initial list specified 42 reporting elements. We acknowledge that other researchers may identify additional, distinct elements that would extend this list. We selected 26 “essential” elements to generate a practical, usable tool but we would recommend that researchers describing GB-BCIs consider whether any of the remaining 16 elements are relevant to their particular intervention. Our checklist is designed to specify a minimum set of descriptions essential to replication of any group-based intervention but particular interventions may require additional elements to ensure accurate replication.

To ensure comprehensiveness of description of interventions, the checklist should be used alongside other relevant reporting guidelines, such as CONSORT [14], CONSORT-SPI for social and psychological interventions [19], GREET for educational interventions [58] or TIDieR checklist [20]. For example, when describing a RCT of a group-based behaviour-change intervention, the authors should use the CONSORT guidelines to ensure comprehensive reporting of the study, the TIDieR checklist for reporting of the intervention elements, and our checklist to ensure comprehensive reporting of group-based delivery. Reporting guidelines that are relevant to a particular study or intervention type can be identified by searching a database of reporting guidelines on the EQUATOR Network’s website (www.equator-network.org) [22]. Relevance is critical as applying irrelevant or poorly specified guidelines, checklists or taxonomies may undermine accurate reporting [59]. Some of the reporting elements in our checklist, such as details of the contact time, activities, materials, setting, participants and providers are similar to elements included in other reporting guidelines. However, the definitions of elements in our checklist extend those provided elsewhere by specifying what, in particular, is needed to describe *group-based* interventions. At the same time many other elements, such as sequencing of sessions, group composition, methods for group allocation, facilitators’ group assignment, facilitators’ training in and style of group facilitation, are unique to group-based interventions and have not been included in other guidelines. Consequently, the group-focused nature of our guidelines, together with the greater specificity of reporting guidance offered, allows our checklist to be used in tandem with and independently from other tools by designers and researchers of group-based interventions. Moreover, it can be used not only as a guideline for reporting but also as a checklist of important elements when designing group interventions and as a tool for assessing the quality of reporting of GB-BCIs.

Finally, we acknowledge that a lack of consensus exercise, such as the Delphi exercise, is a major limitation of the proposed checklist as a reporting guideline. However, the checklist was intended as preliminary work and extension of the earlier framework for design and delivery of group interventions [40] by providing a more practical tool to improve the reporting quality and enable a reliable assessment of reporting quality. Therefore, considering limited time and resources, a full-scale consensus exercise was not conducted but could be undertaken in the future to develop the checklist further.

### Implications

Current reporting of GB-BCIs in scientific papers often prohibits accurate replication of interventions and identification of their ‘active ingredients’. If evaluated interventions cannot be compared and replicated, then behavioural science cannot advance in a cumulative fashion. Imagine a chemist who creates a new compound that is observed to exhibit desirable capacities but fails to keep adequate laboratory notes so that other chemists have to guess and approximate the compound design to reproduce the observed effects. Such imprecision and parallel re-invention undermines optimization of limited research funds and is strongly discouraged in natural science. Yet such imprecision appears to be the norm in scientific reporting of GB-BCIs. We found that many evaluation papers do not even specify that an evaluated intervention was delivered using group-based sessions in either the title or abstract making it difficult for reviewers to even find GB-BCI evaluations.

We also found that the reasons for delivering the intervention using a group were very rarely reported. Groups may be used to save time and reduce costs, to invoke change processes found to follow from particular group dynamics in previous research or to create social support for participants. Whatever the reason, this has implications for decisions about group size, group activities and group facilitation. Designing GB-BCIs without consideration of previous research on group dynamics and personal change and social support processes ignores decades of relevant research.

While some reporting elements are routinely reported, such as the number of group sessions, duration of the intervention, and facilitators’ professional characteristics, others, such as room configuration and continuity of participants’ group membership, are rarely mentioned. Thus, the comprehensiveness of GB-BCI descriptions varies widely across scientific papers. For example, across the 55 papers coded to test our comprehension and reliable use of our checklist, we found one paper that reported only one of the 26 specified elements and one paper that reported 25 of the 26 elements. Clearly, standardization of reporting practice could greatly enhance behavioural scientists’ capacity to understand research involving GB-BCIs and practitioners’ ability to implement this work to enhance healthcare delivery.

The checklist and longer list of reporting elements were designed to ensure comprehensive intervention descriptions. However, when intervention descriptions are sufficient to allow identification of any one of the specified reporting elements, they may still vary in regard to the depth and clarity with which they describe that element. Consequently, two separate intervention descriptions that both include all specified reporting elements could still differ substantially in the ease with which they can be accurately replicated. Thus, while comprehensiveness of reporting is a prerequisite to accurate replication and can be encouraged and supported through use of checklists, such as that reported here, authors and editors also need to ensure that the depth of description is sufficient to allow accurate translation of intervention elements into real-life replication.

The restrictive effect of journal word limits on intervention descriptions cease to be relevant as journals move to publishing online and providing opportunities for substantial supplementary documents to be made accessible alongside scientific papers [60]. Including intervention manuals alongside evaluation reports would allow scientists to understand the nature of an intervention by reading the scientific report and simultaneously facilitate accurate replication using the more detailed manual. Unfortunately, despite calls to editors to insist on publication of behaviour change intervention manuals alongside evaluation reports, as included in the Workgroup for Intervention Development and Evaluation Research (WIDER) reporting recommendations [61, 62] and good examples being set by some journals [63], it is often very difficult to access manuals of behaviour change interventions that allow exact replication.

Interventions may be developed in stages or may involve adaptation of existing programmes. In these cases, detailed intervention descriptions may be included in previously published protocols, reports of intervention development or evaluation reports. Such publications should be clearly referenced so that the most comprehensive description can be accessed. Moreover, when implementation of an intervention differs from a previous description, between groups receiving the intervention, or between delivery stages, for example, in relation to the type of venue, or the number, length or frequency of sessions, these variations must be specified if the (potentially effective) intervention is to be replicated with fidelity. For example, if the length of sessions varied, the authors should clearly indicate that providing the range and mean time that the groups received (e.g. ‘The three groups received four sessions of 30 to 45 min long (mean 39 min), but the length of sessions varied between groups: in total, group A received 135 min, group B received 160 min, and group C received 170 min’; ‘the first group session was 1.5 h and the remaining five sessions were 1 h long’). This should be followed by reporting of the reasons why such differences occurred, for example, due to practical reasons (e.g. venue accessibility, holiday period), or whether they were designed as part of the intervention testing (e.g. comparing shorter with longer sessions). Failure to describe such variations (e.g., an increased numbers of sessions) could mean that replications test intervention variants that are importantly different in their effects on target behaviours.

Finally, it is worth noting that the literature includes examples of good reporting practice. Some papers provide comprehensive descriptions of GB-BCIs, others refer to available manuals, and some include examples of tables being used effectively to summarize details on session content, materials, change techniques and theoretical constructs [64–66], and diagrams to summarize contact time in groups [67]. Comprehensive description of GB-BCIs can be undertaken within current resources. Editorial guidance using standardized easy to use checklists, such as that presented here, could quickly accelerate the advance of behavioural science in this area [68].

## Conclusion

We defined a set of 42 reporting elements that could enhance the comprehensiveness of descriptions of GB-BCIs. We reduced this to a checklist of 26 essential reporting elements and showed that this could be used to reliability identify whether or not these elements are included in intervention descriptions. The checklist (and accompanying coder manual) is a practical tool that can be applied to all group-based behaviour-change interventions to improve the quality of reporting and to check the comprehensiveness of intervention reporting, and, thereby, facilitate accurate replication. It can also be used as a preliminary “taxonomy” of design elements of GB-BCIs and be used in planning and designing group interventions. As such it may be of use to a wide range of intervention developers, authors, reviewers and editors.

## Additional files

Additional file 1:
**Extensive list of reporting elements for group-based behaviour change interventions.** Additional file 1 includes a table with an extensive list of 42 reporting elements important for replicating and comprehensive reporting of group-based behaviour-change interventions (identified in response to the Research Question 1). (PDF 518 kb) Additional file 2:
**Coder Manual for Identification of Reporting Elements in Reports of Group-Based Behaviour-Change Interventions (GB-BCIs).** Additional file 2 includes a coder manual with coding guidelines, suggestions and examples to facilitate reliable coding of the content of reports of GB-BCIs. (PDF 706 kb)
