# Supplementary material for: A checklist to improve reporting of group-based behaviour-change interventions
Source: BMC Public Health. 2015 Sep 25;15:963. doi: 10.1186/s12889-015-2300-6 (PMC4583168; doi:10.1186/s12889-015-2300-6)
Supplement: Additional file 1: — Extensive list of reporting elements for group-based behaviour change interventions. Additional file 1 includes a table with an extensive list of 42 reporting elements important for replicating and comprehensive reporting of group-based behaviour-change interventions (identified in response to the Research Question 1). (PDF 518 kb) [file 12889_2015_2300_MOESM1_ESM.pdf]

**Additional file 1:**

## Extensive list of reporting elements for group-based behaviour change interventions

In the process of selecting reporting elements we recognized that not all of them might be relevant to all group interventions. Therefore, in the reporting checklist (Table 1 in Borek et al.) we included only these elements that we expect to be common to all group-based behaviour change interventions. These elements constitute, in our view, the minimum content of the reports of group-based health interventions. However, there are also other elements that should be included in the comprehensive reports of group-based interventions but might not apply to all group-based interventions. The table below includes all the reporting elements from the checklist *and* the additional reporting elements that the authors should consider when describing a group-based intervention.

Reporting elements in **bold** font are included in the checklist and the number in brackets ( ) corresponds with the numbering of the elements in the checklist (see Table 1 in Borek et al.).

**Table 1. Extensive list of reporting elements for GB-BCIs.**

| **Reporting Elements** | **Example Questions Addressed**<br>*(When applicable)* |
|---|---|
| **INTERVENTION DESIGN** | |
| 1. Labelling of group-based intervention in title or abstract | Is the group-based delivery mode clearly indicated in the title and/or abstract? |
| 2. Rationale for group format | Why was the group mode of delivery selected?<br>What was intended to be achieved by using groups as opposed to alternative formats?<br>To what extent were the advantages and disadvantages of using groups considered? |
| **3. (1) Intervention source or development methods** | What is the source or origin of the intervention?<br>What materials, research methods or models were used in the process of developing the intervention?<br>Who was consulted in this process? |
| 4. Adaptations for group setting | How was the design of the program adapted specifically for group setting? |
| 5. Other modes of delivery | What other modes of delivery, if any, were used in addition to groups?<br>How were the other delivery formats used? |
| **6. (2) General** | Where were the sessions delivered? |

| | |
|---|---|
| **setting** | What was the setting like (e.g., any characteristics of the institution)?<br>Why was it selected?<br>What was the wider environmental and cultural setting in which the intervention was delivered? |
| **7. (3) Venue characteristics** | What was the venue where the sessions were delivered like?<br>What was the room setting where the groups met like?<br>Was the room setting or venue purposefully manipulated for the sessions (e.g., seating arranged to encourage interaction, to facilitate learning)?<br>Where there any practical issues related to the room setting or venue (e.g., proximity, parking spaces, access issues)? |
| **8. (4) Total number of group sessions** | How many group sessions were delivered in the intervention?<br>Did all the groups receive the same number of sessions? |
| **9. (5) Length of group sessions** | How long were the sessions? (Range, mean) |
| **10. (6) Frequency of group sessions** | How often were the sessions delivered?<br>Was the frequency of sessions the same or changing throughout the duration of the intervention? |
| **11. (7) Duration of the intervention** | Over what period of time were the sessions delivered? |
| 12. Follow-up sessions | Were any follow-up or maintenance sessions delivered after the core part of the intervention?<br>If yes, how did they differ from the core sessions? |

| | |
|---|---|
| 13. Additional contact time | What other pre- and post- intervention contact did the participants have? |
| 14. Total contact time | How much contact time (in total) in groups did the participants receive in the intervention? |
| **INTERVENTION CONTENT** | |
| **15. (8) Change mechanisms or theories of change** | How was the intervention intended to work?<br>What mechanisms or theories of change was the intervention based on?<br>How were the mechanisms of change incorporated in the intervention design and/or delivery? |
| **16. (9) Change techniques** | What techniques were used in group sessions to prompt (behaviour / cognitive) change? |
| **17. (10) Session content** | What were the group sessions about?<br>What was the focus and thematic content of the sessions?<br>Was different session content planned for each session?<br>Was the content of the sessions fixed or flexible (i.e., to what extent the content was pre-determined or influenced by the participants and facilitators)? |
| **18. (11) Sequencing of sessions** | Was the content of the sessions delivered in a logical sequence following the process model?<br>Were the later sessions based on or related to the content of the earlier sessions, i.e., was the session content progressive or repetitive?<br>How was the sequence of the sessions designed?<br>Were the participants expected to attend all of the sessions in the |

| | |
|---|---|
| | intended order? |
| **19. (12) Participants' materials** | What materials (e.g., booklets, newsletters) or tools (e.g., pedometers) did the participants use during and outside the sessions?<br>How were these materials developed and what they included?<br>How were these materials and tools used and incorporated in the sessions? |
| **20. (13) Activities during the sessions** | What activities did the participants and facilitators engage in during the sessions, i.e., what did they do during the sessions?<br>What was the structure of the sessions? |
| 21. Activities outside the sessions | What, if any, activities were organized or offered outside the group sessions? |
| 22. Additional incentives or rewards | Were any additional methods used to encourage attendance at the sessions and adherence (e.g., financial incentives, giveaways, reminders)? |
| **23. (14) Methods for checking fidelity of delivery** | What methods were used to check the fidelity of delivery of intervention content, i.e., if the intervention was delivered as intended? |
| 24. Participants' feedback | Were the participants asked to provide feedback on the intervention and session delivery?<br>What was the participants' feedback?<br>How was the received feedback used? |
| **PARTICIPANTS** | |
| 25. Recruitment | How were the participants recruited to the program?<br>Were there any requirements to be met before enrolling (e.g., attending |

| | |
|---|---|
| | an induction session, keeping food/physical activity diaries, were there any entry "rituals")?<br>Was the access to the intervention restricted or open? |
| 26. Waiting time between recruitment and intervention beginning | How much time passed between the recruitment of participants and the delivery of the first session? |
| 27. Number of groups | How many different groups were formed within the intervention arm? |
| **28. (15) Group composition** | Who were the participants in the groups (e.g., in terms of participants' gender, age, ethnicity, religion, health condition etc.)?<br>Were there any important differences between the groups in participants' characteristics or were participants' characteristics representative of the whole sample?<br>Did the participants' "significant others" attend the groups? |
| **29. (16) Methods for group allocation** | How were the participants allocated to different groups (e.g., self-selected groups or allocated to groups)?<br>Were the groups purposefully or opportunistically composed? |
| **30. (17) Continuity of participants' group membership** | Could the participants attend different groups or were they allocated to one group throughout the intervention (i.e., open or closed access to groups)?<br>Did the participants attend the sessions with the same participants? |
| **31. (18) Group size** | How many participants were in the groups? (Range, mean) |

| | |
|---|---|
| | Was the actual group size as intended?<br>Why was this group size selected? |
| **FACILITATORS** | |
| **32. (19) Number of facilitators** | How many facilitators delivered the sessions, i.e., did the same facilitator(s) deliver whole sessions or were parts of the sessions delivered by different facilitators? (I.e., this refers to the number of facilitators within sessions.) |
| **33. (20) Continuity of facilitators' group allocation** | Did the same facilitator(s) deliver all of the sessions to the same group(s) or did the facilitators change between the sessions? (I.e., this refers to the number of facilitators between sessions.) |
| 34. Facilitators' allocation to the intervention groups | Did the same or different facilitator(s) deliver the sessions to different groups of participants in the intervention? (I.e., this refers to the number of facilitators in the intervention.) |
| **35. (21) Facilitators' professional background** | Who were the facilitators in terms of professional background?<br>What relevant professional qualifications, skills, knowledge and experience did the facilitators have? |
| **36. (22) Facilitators' personal characteristics** | Who were the facilitators in terms of age, gender, ethnicity etc.?<br>To what extent were the facilitators' characteristics similar to or different from the participants' characteristics?<br>Could the participants identify themselves with the facilitators?<br>What other, relevant but not professional, experience and competences did they have (e.g., cultural competence, prior participation in the program)? |

| | |
|---|---|
| **37. (23) Facilitators' training in intervention delivery** | What training did the facilitators receive in delivering the intervention?<br>Who provided the training?<br>What did the training include?<br>How long was it?<br>Development of what skills and knowledge related to the intervention was intended in the training? |
| **38. (24) Facilitators' training in group facilitation** | What training did the facilitators receive in group facilitation methods?<br>Who provided the training?<br>What did the training include?<br>How long was it?<br>Development of what skills related to group facilitation was intended in the training? |
| 39. Support for the facilitators | Were the facilitators offered any support during the delivery of the intervention (e.g., meetings with other facilitators, contact with intervention designers)? |
| **40. (25) Facilitators' materials** | How were the facilitators instructed to deliver the sessions?<br>What materials and tools were the facilitators provided with to deliver the sessions?<br>To what extent was the session delivery standardized by the use of a manual or script?<br>What teaching aids did the facilitators use (e.g., presentation slides, flip charts)? |
| **41. (26) Intended facilitation style** | What was the intended facilitation style (e.g., didactic, interactive)?<br>What techniques or approaches did the facilitators use to achieve the |

| | |
|---|---|
| | intended facilitation style?<br>What was the intended role of the person delivering the group sessions?<br>To what extent were components of the intervention tailored towards individual participants? |
| 42. Group processes | How did the groups work together?<br>What group processes were purposefully activated in the groups (e.g., social identification, social comparisons, peer support)?<br>How were they activated?<br>What was the interaction between the participants and facilitators like?<br>What was the interaction between the participants in the group like?<br>Was any inter-group interaction encouraged (e.g., inter-group competition)? |
