# Supplementary material for: A checklist to improve reporting of group-based behaviour-change interventions
Source: BMC Public Health. 2015 Sep 25;15:963. doi: 10.1186/s12889-015-2300-6 (PMC4583168; doi:10.1186/s12889-015-2300-6)
Supplement: Additional file 2: — Coder Manual for Identification of Reporting Elements in Reports of Group-Based Behaviour-Change Interventions (GB-BCIs). Additional file 2 includes a coder manual with coding guidelines, suggestions and examples to facilitate reliable coding of the content of reports of GB-BCIs. (PDF 706 kb) [file 12889_2015_2300_MOESM2_ESM.pdf]

Additional file 2:

Coder Manual for Identification of Reporting Elements in Reports of Group-Based Behaviour-Change Interventions (GB-BCIs)

As specified in Borek et al.'s *A Checklist to Improve Reporting of Group-Based Behaviour-Change Interventions*

Introduction

The checklist for group-based behaviour-change interventions (GB-BCIs) can be used both as (1) guidance for reporting of group-based interventions, and (2) as a tool for assessing the quality of reporting in published articles. This coder manual includes guidelines for coding when using the checklist to assess reporting quality.

Our intention is not to generate total scores quantifying the quality of articles. We advise making cautious judgments about the overall quality of reports based on the calculations of 'scores' because the reporting elements have unequal weights.

When using the checklist as an assessment tool, it is important to remember that even when an element is not reported, it may have been delivered in the intervention; the omission may be in reporting rather than delivery.

The checklist does not allow comparisons to be made between articles in the extent and quality of descriptions, since it only indicates whether a particular reporting element is present or absent. For example, in one article the authors might only state that the facilitators

were trained in delivering the manual-based intervention whereas in another the authors might provide a more extended description of the content, duration and who provided the training etc. In both cases, however, the reporting element '*Facilitators' training in intervention delivery*' would simply be coded as '*present*'. Hence, to assess quality of intervention descriptions requires a judgment about whether the level of description is sufficient to allow replication, as well as the identification of simple presence vs absence of the elements of description suggested here.

We propose that the checklist is used as a basis for identifying strengths and deficiencies in descriptions of group-based interventions. It can be used in systematic reviews to compare all included articles in a systematic and reliable way, to identify major omissions and to enable the assessment of the overall quality of reporting of selected GB-BCIs. It can also be used to identify examples of articles in which group interventions were reported particularly well.

To ensure consistent use of the relevant terms, we refer to (a) *participants* as people receiving the intervention (in the literature also referred to as 'patients' or 'group members'), (b) *facilitators* as people delivering group sessions in the intervention (also referred to as 'group leaders'), (c) *groups* as groups of two or more participants, and (d) *(group) sessions* as meetings of participants with at least one facilitator.

Below are the coding guidelines that we developed in the process of testing and revising the checklist, and that should facilitate reliable coding.

General coding guidelines

1. **Never infer that the element is reported.** Unless you can identify the text that provides an unambiguous description you should not code reporting elements as 'present'. For example, even though it might be possible to infer that the participants were randomly allocated to the groups, or that the group composition was a representative sample of the intervention participants, you should not code it as 'present' unless it is clearly stated in the text.
2. **Only code the information that refers to the intervention.** Always ensure that text relating to the checklist's reporting elements refers to the intervention being assessed. For example, there might be a discussion about theories of behaviour change in the introduction to the article but this should not be coded unless the description explains that these theories shaped the intervention.
3. **Apply the checklist to each group-based intervention separately.** Where more than one different group-based intervention is delivered in a trial code the information that refers to each group-based intervention separately. However, if the differences between group-based interventions relate to the content only (e.g., different dietary recommendations or change techniques used) and not to the format or structure of the groups (e.g., number or frequency of sessions, delivery style) then the interventions can be coded as one.
4. **Code only clear and meaningful descriptions.** Only descriptions which could be used to compare interventions, for example in extracting data for a systematic review, or in replicating the intervention should be coded as 'present'. The question being asked is: would this description be sufficient and meaningful in data extraction for a systematic review? Overly generic or ambiguous descriptions (e.g., facilitators described as 'staff

members', intervention as 'theoretically-based') that leave the reader with little understanding of how the intervention was actually designed or delivered should not be coded.

Other practical coding tips

5. Familiarize yourself with the checklist by reading and re-reading the checklist with descriptions and examples. It is also useful to code a few articles initially, check the coding with another independent coder and discuss any discrepancies and uncertainties trying to identify why they occurred (e.g., misunderstanding or differences in interpretation of coding instructions, information missed in reading) and addressing these issues if possible (e.g., coming to an agreed interpretation, re-reading coding instructions, reading articles more carefully).
6. To minimize the risk of making inferences, the checklist reporting elements should be linked to particular fragments of text. This can be done by underlying the text and writing the name or number of the reporting element in the margin or by highlighting the text and inserting comments linked to the text (e.g. in a Word or pdf document). Software for qualitative coding can be also used effectively with the advantage of having all the references to the same reporting element at hand should this be required, for example, in writing up findings from this exercise as part of a review.
7. It is important to decide whether or not a reporting element is present or absent but when one feels that the decision is uncertain this could be qualified by inserting a question mark ('?'), or another agreed symbol. This highlights elements that warrant special discussion with another coder. It may also be useful to identify and highlight particularly good descriptions by inserting a plus ('+'), or another agreed symbol, next to very good and comprehensive descriptions.

8. Coding of the same fragment of text in relation to more than one reporting element may occur (e.g., writing action plans should be coded both as activities and change techniques) but should be avoided as much as possible. Careful consideration should therefore be given as to whether it is justified and necessary to code something in relation to more than one reporting element.
9. Prior to coding a decision should be made as to whether supplemental information referenced in the articles being coded should be included in the assessment of the reporting quality or not. For example, some might decide that if there are references to relevant details and the sources are publicly available (i.e., available online as supplementary information or included in earlier publications), the coder should include them in the assessment of the quality of reporting.
10. Although most of the reporting elements should appear in the methods section of an article, it is recommended that the full article is reviewed in the process of coding. This is to ensure that reporting elements which are described in other parts of the article are also identified. For example, theoretical background might be discussed in the introduction, group size and room setting might be included in the results sections etc.

Table 1. Coder guidelines for identification of reporting elements of group-based behaviour-change interventions.

Reporting Elements	Description & Coding Suggestions	Examples
**1. Intervention source or development methods**	Describes the source (origin) and/or methods used for developing the intervention.<br><br>A brief reference or simple indication is sufficient.	Reference to another program on which the intervention was based; description of the process of developing an original intervention, e.g., including intervention mapping, PRECEDE-PROCEED model, findings from interviews or focus groups etc.<br><br>*"The Weight-Wise intervention was adapted from the Diabetes Prevention Program (Diabetes Prevention Program Research Group, 2002) and PREMIER lifestyle interventions (Svetkey et al., 2003)."* [1: 391]<br><br>*"We modified the previously developed DPP intervention to be appropriate for Latino individuals at risk for type 2 diabetes. Focus groups [16] were conducted to assess unique knowledge gaps regarding diabetes prevention, attitudes toward prevention, and challenges to weight loss in this population.*

		Additional focus groups were used to pre-test the acceptability of the intervention materials (e.g., soap opera, goal sheets).” [2: 337]
**2. General setting**	Reports the type of setting where the group sessions were delivered.<br><br>Code references to the setting where group sessions took place and not the study setting where, for example, the measurements were taken.<br><br>A simple but clear indication is sufficient.	A community setting, school, university, worksite, health care practice, hospital etc.<br><br>*“Sessions were delivered at the central hospital site where the nutrition clinic was located.”* [3: 109]<br><br>*“Most study activities (i.e., screening, recruitment, group intervention sessions, and follow-up assessments) were held at the Lawrence Senior Center, a centrally located social service facility.”* [2: 336]
**3. Venue characteristics**	Describes the set up or configuration of the room (or other venue) where the group meetings took place.<br><br>A brief description is sufficient.	Sitting arrangements (circle, semi-circle); the type or size of the room (community hall, classroom, lecture theatre); purposeful manipulations to the room setting to facilitate interaction or learning etc.<br><br>*“Tables might interfere with the open group set- up. Space restriction made provision of tables impractical.”* [3: 116]

			"The program was administered at Osaka Prefecture University and City Health Center using classrooms for the groups and also individual consultations." [3: 83]
**4.**	**Total number of group sessions**	The total number of group sessions in the program is reported or it is possible for this to be calculated.	*"The weight loss intervention consisted of 22 group sessions led by nutrition and behavioral counsellors, over 6 months."* [4: 87]
**5.**	**Length of group sessions**	Reports the length of group sessions (average and/or range).	*"The duration of the first group session was 1.5 hours and the remaining group sessions were 1 hour."* [2: 337]
**6.**	**Frequency of group sessions**	Reports the frequency of group sessions, i.e., how often they were delivered.<br><br>Code only specific (quantifiable) information and not vague descriptions, such as, "as needed".	Weekly, monthly, X sessions regularly delivered over Y months etc.<br><br>*"Each weight management group (maximum 12 men) meets weekly over 3 months for twelve 60-minute evening sessions."* [5: 72]
**7.**	**Duration of the intervention**	Reports the duration of the intervention.<br><br>Code information in relation to the period of	*"Participants attended closed group sessions weekly during the intensive phase in the first six months. The less-intensive phase consisted of biweekly meetings for months 7–9 and monthly meetings for*

	time over which the group sessions were delivered and not the duration of the study (up to the last follow-up measurement).	*months 10–12."* [6: 151]<br><br>*"The health education intervention was received during the first 4 weeks."* [7: 426]
**8. Change mechanisms or theories of change**	Describes how the intervention was intended to work by identifying change mechanisms or under-pinning theories of behaviour change.<br><br>It is not sufficient to generally review theories or use a theory to explain change processes post-hoc as part of an evaluation of the intervention. Code only descriptions of theories or mechanisms of change that are directly linked to the intervention, i.e., that are described as being used as a basis for the intervention.	Health belief model, theory of planned behaviour, empowerment, self-efficacy etc.<br><br>*"The intervention approach, based on social cognitive theory, behavioral self-management techniques, trans-theoretical, or stages-of-change, models and motivation enhancement [27–31] was designed to be supportive, participant-centered and interactive."* [4: 88]
**9. Change**	Describes the techniques	Encourage self-disclosure, provide

**techniques**	used in group sessions to prompt change. These may be derived from the mechanisms or theories of change on which the intervention was based (see above), and may or may not use established taxonomies of behaviour change techniques [e.g., 8, 9].<br><br>Code only techniques that are explicitly named, i.e., can be linked to particular fragments of text.<br><br>This may also include sets of techniques, such as motivational interviewing, and techniques that do not target behaviours directly, e.g., stress management.	information about behaviour – health link, prompt goal setting and goal review, provide encouragement, provide feedback on performance etc.<br><br>*"The intervention content utilized BCTs [behaviour change techniques] found to be associated with intervention effectiveness (Table 1)… For example, the BCTs of intention formation and behavioral self-monitoring were presented initially, followed by specific goal setting and barrier identification in subsequent sessions – concluding with relapse prevention (Table 1)."* [10: 109]<br><br>*"Guided small-group activities followed the check-in and fostered problem solving, social support and relapse prevention planning. Group activities included calorie awareness activities, record-keeping skill development, nutrition and physical activity demonstrations and goal-setting modules. At the end of each session, each participant developed a plan for the upcoming week by*

		setting food and exercise goals, and a specific action plan for achieving each goal." [4: 88]
**10. Session content**	Describes the content of the sessions in terms of themes or topics covered; i.e., what the sessions were about.<br><br>This can be described either generally or separately for each session.<br><br>Note that the description should be related to the thematic content of the group sessions and not just to the overall content or targets of the intervention.	Health problems related to obesity, process of behaviour change, healthy lifestyle, dietary and physical activity recommendations etc.<br><br>*"Table 1 provides detailed information on session titles, content focus, and learning basis of the DPP-adapted curriculum [20]."* [11: 308-309]<br><br>*"Intervention content was topic driven (nutrition, physical activity, psychology) and theory driven (problem solving, stimulus control, managing high-risk situations, skill building, reinforcement, self-monitoring, developing social support, identifying pros/cons, and self-efficacy) [16]... Topics included lifestyle change, portion estimation, finding the fat, meeting dietary needs according to MyPyramid, activity adoption and maintenance, progressive relaxation and deep breathing*

		for stress management, supportive environments, emotional eating, social support, and maintaining behavior change."[12: S53-S54]
**11. Sequencing of sessions**	Indicates whether there is a logical (sequential) progression of session content or, alternatively, that the content of all sessions is the same, i.e., a repetitive , or "rolling", program with no particular start or end point.<br><br>Note that it can be assumed that the content is sequential (code as 'present') if the topics and/or content are described separately for each session, e.g., in a table.	Indicates that the content of the session is progressive or repetitive; describes the latter sessions as based on or related to the content covered in the earlier sessions etc.<br><br>*"The first session covered diabetes-related definitions and the importance of physical activity. Subsequent sessions promoted eating more vegetables and fruits and less saturated fat and added sugar, setting goals, getting social support for behavior change, and maintaining behavior change."* [13: 3]<br><br>*"The content was sequential, with each session building on the foundation achieved in the previous sessions."* [3: 84]
**12. Participants' materials**	Reports what materials or tools the participants used during and outside the group sessions.	Participant's manual, information sheets, food and physical activity diaries, pedometers, weighing scales or other equipment, newsletters etc.

	This refers to the materials that were provided for the participants by the intervention staff.<br><br>Note that a generic description, such as 'intervention materials', is insufficient.	*"Two educational booklets, one was on menopause and CVD, the second one was on diet were prepared according to the updated American guideline for prevention of CVD in sedentary postmenopausal women. Participants in the diet group received these educational booklets."* [14: 523]<br><br>*"…all participants received GLB participant handouts, weekly self-monitoring booklets, a fat- and calorie-tracking book, and a pedometer."* [15: 662]
**13. Activities during the sessions**	Describes what the participants and the facilitators did during group sessions, i.e., what happened *during* the sessions.<br><br>Note that these activities might overlap with the change techniques (see above) but may also include descriptions of how change	Discussing pre-sessional reading, small group learning activities, discussions, writing and reviewing action plans, group exercise, cooking demonstrations, lectures or presentations from the facilitators etc.<br><br>*"Each session began with a check-in focused on accomplishments and barriers with regard to each participant's individual weekly goals. Guided small-group activities followed the check-in and fostered problem solving, social support and relapse*

	techniques (e.g., providing information, providing social support, prompting action planning) were used in the sessions.	*prevention planning. Group activities included calorie awareness activities, record-keeping skill development, nutrition and physical activity demonstrations and goal-setting modules. At the end of each session, each participant developed a plan for the upcoming week by setting food and exercise goals, and a specific action plan for achieving each goal… The intervention leaders reviewed the diaries each week and provided encouragement and support for keeping records. Participants were weighed at each group session.”* [4: 88]<br><br>*“Each class consisted of lectures… presented by either a physician… or registered dietitians and physical trainers, while some classes… involved group support education, group dynamics and individual consultations. Dietary skills taught by dietitians included portion control, eating order (vegetables first and carbohydrates last) and glycemic index, dental care by dentists, and practical day-to-day exercises by physical trainers… In*

		particular, the importance of diet self-management was emphasized during two cooking classes held in the first and fifth months of the education period… meal planning or exercise plans and were expressed and discussed with group members during every class. Educators of each group wrote and gave feedback during all the classes, after monitoring the diaries of each participant…" [3: 83-84]
**14. Methods for checking fidelity of delivery**	Reports methods used to check the fidelity of intervention delivery, i.e., methods used to check if the sessions were delivered as designed.<br><br>Note that the methods for *checking* fidelity of delivery (e.g., session recordings, observations) may be different to methods for *ensuring* fidelity (e.g., facilitators' training, manual). Only code	Session recordings, observations, checklists completed after the sessions etc.<br><br>*"Group leaders met together weekly to discuss the progress of each group and to assure the topics were covered in similar ways. To ensure that leaders conducted groups according to the session outlines, we used a system of random observations. Observers (lead interventionist, principal investigator, co- investigators and other outside experts) completed an observation checklist and discussed any findings with the group leader in a debrief session. The principal investigator regularly reviewed*

	descriptions of methods used for checking the fidelity of group delivery.	*the observation checklists."* [4: 88]<br><br>*"For process evaluation, CSREES educators completed a brief weekly questionnaire about attendance, participant engagement with the classes, and ability to carry out the intervention as designed. In addition, study personnel observed 1 class at each site to assess fidelity to the intervention."* [16: 1273]
**15. Group composition**	Provides information on the composition of the groups in the intervention, i.e., who were the participants in the groups or whether there were any differences in the participants' characteristics between groups.<br><br>This is related to the characteristics of the participants in the groups and not just to the general characteristics of the study participants.	Single-gendered groups, groups with people of similar ethnicity or age, groups representative of intervention participants, groups with participants' 'significant others'.<br><br>*"Same-sex groups were requested by about 20% of participants… Given the central role of the family, we actively sought family support by inviting family members to attend all sessions and activities… Participants who agreed to the lifestyle intervention were allocated to groups of their choice; either mixed gender or all female."* [11: 309-310]

**16. Methods for group allocation**	Describes methods used to allocate the participants to different groups.	Participants could select a group to attend; participants were purposefully allocated to different groups by the intervention staff; participants were allocated to groups as they were recruited (opportunistically).<br><br>*"Patients providing written consent were referred to the research team to schedule attendance at the first intervention session."* [3: 109]<br><br>*"The 34 eligible and interested participants each selected one of two weekday evening meeting times for which they were available, and then the group meeting times were cluster randomized to either the Catholic-Tailored (n = 17) or the Standard Behavioral (n = 17) condition."* [17: 383]
**17. Continuity of participants' group membership**	Indicates whether there was continuity in participants' membership in a group throughout the program or if participants could attend different groups.	Closed / open group sessions; participants could attend group meetings at different times/days etc.<br><br>*"Participants attended closed group sessions weekly during the intensive phase*

		in the first six months.” [6: 153]
**18. Group size**	Reports the number of participants per group (average and/or range).<br><br>Only code quantifiable information, i.e., the number of participants (average/range), and do not code general descriptions, such as ‘small group’.	*“Each group (20–25 participants) met weekly for 90min.”* [4: 88]<br><br>*“Group size was set at eight to ten members in order that the sessions were manageable without diminishing the opportunity for expression and individualization within each group.”* [18: 83]
**19. Number of facilitators**	Reports the number of facilitators delivering the sessions, i.e., how many facilitators delivered each of the sessions.<br><br>Note that this refers to the number of facilitators per session not the overall number of facilitators in the intervention (i.e., team size).	*“Sessions lasted 90 min and were delivered by one researcher with 7 years of experience…”* [19: 3]
**20. Continuity of facilitators’**	Indicates whether there was continuity in facilitator’s	*“The trained study nurse facilitated all core-curriculum sessions.”* [11: 310]

**group assignment**	assignment to a group throughout the intervention, i.e., if the same or different facilitator(s) delivered the sessions to the same group of participants.	*"For each phase of the intervention, the same registered dietitian and clinical psychologist were in charge of the group."* [20: 959]
**21. Facilitators' professional background**	Reports facilitators' professional background, status as a non-professional or qualifications.<br><br>Only code specific references to the profession or other status (e.g. as a lay person, peer), including relevant qualifications or specializations, and do not code generic descriptions, such as 'staff', 'providers', 'experienced / trained in delivering the intervention'.	Health care professionals, dieticians, nutritionists, physical activity trainers, psychologists, health educators, researchers, lay leaders, community members, peer educators etc.<br><br>*"Each site is an American Diabetes Association–recognized DSME program, with nurse and dietitian diabetes educators who participated in this project. All but one of the educators was a certified diabetes educator."* [15: 661]<br><br>*"…health educators from the study staff taught the curriculum… with the assistance of church lay leaders."* [21: 71]
**22. Facilitators' personal characteristics**	Reports relevant personal characteristics of the facilitators, i.e., who they	*"A female research nurse…"* [3: 209]<br><br>*"All study-related procedures were carried*

	were in terms of age, gender, ethnic or cultural background, education level, socio-economic status etc.	*out by trained bilingual personnel who were racially and ethnically identified with the community."* [11: 308]
**23. Facilitators' training in intervention delivery**	Reports what training facilitators were provided with in delivering the intervention.<br><br>A simple and clear indication is sufficient.	*"The nurse received training (1.5 days in duration) on how to deliver the manual-based intervention."* [3: 109]<br><br>*"The PREDIMED food and nutrition professionals… Each was a registered dietitian (RD) trained and certified to deliver the PREDIMED intervention protocol."* [22: 1136]
**24. Facilitators' training in group facilitation**	Reports what training the facilitators were provided with in group facilitation methods, i.e., how to work with and facilitate groups.<br><br>A simple and clear indication is sufficient. Also code references to training of facilitators in communication,	*"One component of the [training] workshop focuses on leading groups…"* [15: 661]<br><br>*"…training in motivational counseling and group management skills. The training included role-playing and mock intervention sessions and was led by a behavioral psychologist and a senior registered dietitian, who also provided ongoing supervision."* [2: 338]

	interpersonal or counselling skills, e.g., motivational interviewing / counselling.	
**25. Facilitators' materials**	Reports whether the facilitators were provided with materials and/or written instructions to be used to guide delivery of the sessions.<br><br>This relates to the materials or written instructions designed specifically for the facilitators, and other than verbal instructions included in the training in intervention delivery (see above) or in the general intervention protocol.	Intervention manual, script for the sessions, discussion guides, presentation slides etc.<br><br>*"Group leaders followed standardized session outlines."* [4: 88]<br><br>*"…the educators… were provided with a taped DVD series of the GLB to allow for review before delivery of the program as needed."* [15: 661]
**26. Intended facilitation style**	Describes the intended style of, or approach for, the session delivery and group facilitation.<br><br>This refers to any	Didactic sessions, lectures, presentation-based, interactive sessions, discussion-based, collaborative, participant-centered, supportive, encouraging; descriptions of group processes and group atmosphere; use of humor to facilitate positive group

	descriptions indicating whether the sessions were interactive or didactic, to what extent individual tailoring of ideas or discussion should be incorporated, and/or what techniques were used to achieve a particular facilitation style or group atmosphere.<br><br>A brief description is sufficient.	atmosphere etc.<br><br>*"The intervention approach… was designed to be supportive, participant-centered and interactive."* [4: 88]<br><br>*"Finally, the use of humor is perhaps most valuable in forging relationships between group members [41] and allowing the men to raise sensitive issues that they might otherwise find difficult or embarrassing to discuss [42,43]."* [5: 73]

References

1. Gustafson A, Khavjou O, Stearns SC, et al. Cost-effectiveness of a behavioral weight loss intervention for low-income women: The Weight-Wise Program. *Prev Med*. 2009;49(5):390-395. doi:10.1016/j.ypmed.2009.09.007.

2. Ockene IS, Tellez TL, Rosal MC, et al. Outcomes of a Latino community-based intervention for the prevention of diabetes: the Lawrence Latino Diabetes Prevention Project. *Am J Public Health*. 2012;102(2):336–342. doi: 10.2105/AJPH.2011.300357.

3. West R, Walia A, Hyder N, Shahab L, Michie S. Behavior change techniques used by the English Stop Smoking Services and their associations with short-term quit outcomes. *Nicotine Tob Res*. 2011;12(7):742-747. doi:10.1093/ntr/ntr120.

4. Elder CR, Gullion CM, Funk KL, DeBar LL, Lindberg NM, Stevens VJ. Impact of sleep, screen time, depression and stress on weight change in the intensive weight loss phase of the LIFE study. *Int J Obes*. 2012;36(1):86–92. doi:10.1038/ijo.2011.60.

5. Gray CM, Anderson AS, Clarke AM, et al. Addressing male obesity: an evaluation of a group-based weight management intervention for Scottish men. *J Mens Health*. 2009;6(1):70–81. doi: 10.1016/j.jomh.2008.11.002.

6. Acharya SD, Elci OU, Sereika SM, et al. Adherence to a behavioral weight loss treatment program enhances weight loss and improvements in biomarkers. *Patient Prefer Adherence*. 2009;3:151-160.

7. Merrill RM, Massey MT, Aldana SG, Greenlaw RL, Diehl HA, Salberg A. C-reactive protein levels according to physical activity and body weight for participants in the coronary health improvement project. *Prev Med*. 2008;46(5):425-430. doi:10.1016/j.ypmed.2007.12.002.

8. Abraham C, Michie S. A taxonomy of behavior change techniques used in interventions. *Health Psychol*. 2008;27(3):379-387. doi:10.1037/0278-6133.27.3.379.

9. Michie S, Ashford S, Sniehotta FF, Dombrowski SU, Bishop A, French DP. A refined taxonomy of behaviour change techniques to help people change their physical activity and healthy eating behaviours: The CALO-RE taxonomy. *Psychol Health*. 2011;26(11):1479-1498. doi:10.1080/08870446.2010.540664.

10. Dombrowski SU, Sniehotta FF, Johnston M, et al. Optimizing acceptability and feasibility of an evidence-based behavioral intervention for obese adults with obesity-related co-morbidities or additional risk factors for co-morbidities: An open-pilot intervention study in secondary care. *Patient Educ Couns*. 2012;87(1):108-119. doi:10.1016/j.pec.2011.08.003.

11. Jaber LA, Pinelli NR, Brown MB, et al. Feasibility of group lifestyle intervention for diabetes prevention in Arab Americans. *Diabetes Res Clin Pract*. 2011;91(3):307-315. doi:10.1016/j.diabres.2010.11.032.

12. Krummel D, Semmens E, MacBride AM, Fisher B. Lessons Learned from the Mothers' Overweight Management Study in 4 West Virginia WIC Offices. *J Nutr Educ Behav*. 2010;42(3):S52-S58. doi:10.1016/j.jneb.2010.02.012.

13. Allen P, Thompson JL, Herman CJ, et al. Impact of Periodic Follow-Up Testing Among Urban American Indian Women With Impaired Fasting Glucose. *Prev Chronic Dis*. 2008;5(3):A76.

14. Abedi P, Lee MHS, Kandiah M, et al. Diet intervention to improve cardiovascular risk factors among Iranian postmenopausal women. *Nutr Res Pract*. 2010;4(6):522-527. doi:10.4162/nrp.2010.4.6.522.

15. Kramer MK, McWilliams JR, Chen H-Y, Siminerio LM. A Community-Based Diabetes Prevention Program: Evaluation of the Group Lifestyle Balance Program Delivered by Diabetes Educators. *Diabetes Educ*. 2011;37(5):659-668. doi:10.1177/0145721711411930.

16. Folta SC, Lichtenstein AH, Seguin RA, Goldberg JP, Kuder JF, Nelson ME. The StrongWomen-Healthy Hearts program: reducing cardiovascular disease risk factors in rural sedentary, overweight, and obese midlife and older women. *Am J Public Health*. 2009;99(7):1271–1277. doi: 10.2105/AJPH.2008.145581.

17. Krukowski RA, Lueders NK, Prewitt TE, Williams DK, Smith West D. Obesity Treatment Tailored for a Catholic Faith Community: A Feasibility Study. *J Health Psychol*. 2010;15(3):382-390. doi:10.1177/1359105309353645.

18. Imai S, Kozai H, Naruse Y, et al. Randomized Controlled Trial of Two Forms of Self-Management Group Education in Japanese People with Impaired Glucose Tolerance. *J Clin Biochem Nutr*. 2008;43(2):82-87. doi:10.3164/jcbn.2008050.

19. Sniehotta FF, Dombrowski SU, Avenell A, et al. Randomised Controlled Feasibility Trial of an Evidence-Informed Behavioural Intervention for Obese Adults with Additional Risk Factors. Verbeek JH, ed. *PLoS ONE*. 2011;6(8):e23040. doi:10.1371/journal.pone.0023040.

20. Provencher V, Bégin C, Tremblay A, Mongeau L, Boivin S, Lemieux S. Short-Term Effects of a "Health-At-Every-Size" Approach on Eating Behaviors and Appetite Ratings. *Obesity*. 2007;15(4):957–966. doi: 10.1038/oby.2007.638.

21. Yanek LR, Becker DM, Moy TF, Gittelsohn J, Koffman DM. Project Joy: faith based cardiovascular health promotion for African American women. *Public Health Rep*. 2001;116(Suppl 1):68-81.

22. Zazpe I, Sanchez-Tainta A, Estruch R, et al. A Large Randomized Individual and Group Intervention Conducted by Registered Dietitians Increased Adherence to Mediterranean-Type Diets: The PREDIMED Study. *J Am Diet Assoc*. 2008;108(7):1134-1144. doi:10.1016/j.jada.2008.04.011.
